# Fall Prevention Shoes Using Camera-Based Line-Laser Obstacle Detection System

**DOI:** 10.1155/2017/8264071

**Published:** 2017-05-15

**Authors:** Tzung-Han Lin, Chi-Yun Yang, Wen-Pin Shih

**Affiliations:** ^1^Graduate Institute of Color and Illumination Technology, National Taiwan University of Science and Technology, No. 43, Sec. 4, Keelung Rd., Taipei 10607, Taiwan; ^2^National Taiwan University, No. 1, Sec. 4, Roosevelt Road, Taipei 10617, Taiwan

## Abstract

Fall prevention is an important issue particularly for the elderly. This paper proposes a camera-based line-laser obstacle detection system to prevent falls in the indoor environment. When obstacles are detected, the system will emit alarm messages to catch the attention of the user. Because the elderly spend a lot of their time at home, the proposed line-laser obstacle detection system is designed mainly for indoor applications. Our obstacle detection system casts a laser line, which passes through a horizontal plane and has a specific height to the ground. A camera, whose optical axis has a specific inclined angle to the plane, will observe the laser pattern to obtain the potential obstacles. Based on this configuration, the distance between the obstacles and the system can be further determined by a perspective transformation called homography. After conducting the experiments, critical parameters of the algorithms can be determined, and the detected obstacles can be classified into different levels of danger, causing the system to send different alarm messages.

## 1. Introduction

When the elderly fall, it can harm their bodies and has serious negative mental impacts on them. In the United States, about 30% of adults above 65 years of age suffer a fall annually [1]. Besides, they bring about a tremendous medical expense in the society. For example, the total cost of fall-related hospitalizations in Texas in 2011 was 3.9 million US dollars. For people aged above 65, the total cost was 2.5 million. As the proportion of the elderly in societies grows, the chances of falls will become larger.

There are many risk factors that can cause the elderly to fall, and they can be roughly classified into three categories: intrinsic factors, extrinsic factors, and exposure to risk [2]. Among the intrinsic factors, different kinds of diseases and reduced physical abilities may increase the probability of falls. As for the extrinsic factors, some studies show that 30% of falls of the elderly are due to accidents and environment-related risk factors [3]. The extrinsic factors include environmental hazards, that is, poor lighting, slippery floors, uneven surfaces, obstacles, unsuitable footwear and clothing, and inappropriate walking aids or assistive devices.

With respect to exposure to risks, Graafmans et al. suggest that the most inactive and the most active people have the highest risk of falls [4]. The most inactive among the elderly may have worse physical capabilities. On the other hands, those who indulge in physical activities can maintain their neuromuscular functioning, which is necessary to keep a balance and to react to falls. However, physical activities also increase greater exposure to environmental risks.

**Figure 1:**
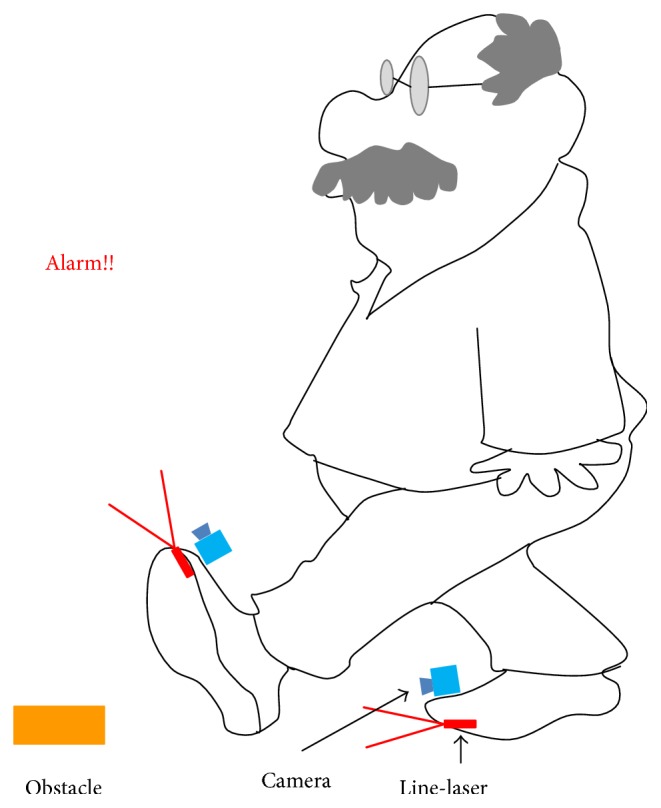
Line-laser obstacle detection system sends alarm messages to catch the attention of the elderly when they encounter dangerous obstacles.

Researchers have worked for a long time to develop various kinds of fall prevention and fall detection systems [5, 6]. Until now, most fall detection systems recognized fall events after they happened. Therefore, a line-laser obstacle detection system that detects environmental obstacles to prevent falls in advance is provided in this paper. The system is installed on the toe end of the shoes, and when the obstacles are recognized, the system will send alarm messages to catch the attention of the elders, as shown in Figure 1. In this manner, the elderly can notice the obstacles that may cause them to fall in advance, thereby reducing the risk of falls. As a result, physical damages and a negative impact on the mental state of the older people can be prevented and the medical costs of the whole society can be saved to a large extent [7].

## 2. Related Work

In order to detect the environmental hazards that may cause falls, related obstacle detection methods and technologies were deeply investigated. Traditionally, obstacle detection has been vital for many mobile robot applications [8]. Popular range-based sensors include ultrasonic sensors, laser range finders, radar, and stereo vision. Each kind of range-based sensor has its limitations. Ultrasonic sensors are cheap but suffer from specular reflection problems. Laser range sensors have higher resolution, but they are more complex and more expensive than ultrasonic sensors. Stereo vision is computationally expensive and difficult to be a practical solution.

Engineers have developed methods and various kinds of systems for obstacle detection based on the abovementioned range sensors. Manduchi et al. implemented a color stereo camera and a single axis radar for cross-country autonomous systems [9]. Zingg et al. presented an approach for wall collision avoidance using a depth map based on optical flow from on board camera images [10]. Oniga and Nedevschi provided a method to classify road surface, traffic isle, and obstacle detection using rectangular digital elevation map from dense stereo data [11]. Zhang et al. developed a novel algorithm for on-road obstacle detection based on stereo cameras [12]. The proposed algorithm in this paper significantly reduces the complexity disparity calculations involved when using a stereo vision technique. In addition, Batavia and Singh developed an obstacle detection methodology, which combines two algorithms: adaptive color segmentation and stereo-based color homography [13]. This algorithm is particularly suited for environments where the terrain is relatively flat and of roughly the same color.

With respect to laser range finders, Fu et al. designed an integrated triangulation laser scanner for obstacle detection installed on miniature mobile robots [14]. The basic components of the triangular laser system are composed of a laser emitter and a camera; therefore, the system becomes smaller and less power demanding. As a result, it is possible to integrate the triangular laser scanners on microrobots.

Stereo vision is, however, computationally expensive. On the contrary, a camera-based line-laser technique that needs much less computation amounts is proposed to detect obstacles in this paper which is a part of Yang's thesis [15]. Because of its lesser computational amount, the extracted line-laser pattern has high potential to be implemented on embedded systems. In addition, the simple components reduce the costs of the overall system. Therefore, a complete integration of the system on shoes for the purpose of fall prevention can be achieved.

## 3. Method

### 3.1. System Configuration

In our system configuration, a line-laser is mounted on the side of the shoes and an RGB camera is fixed but tilted down on the top side of the shoes [16]. The relative position between the line-laser and the camera is extremely rigid to deliver a consistent obstacle detection result. To have a good depth resolution, the distance between these two components should be as large as possible. The tilted angle of the camera can be adjusted according to the detection region in our prototype. Because the average step length of older people is around 0.808 m [17, 18], the detection region of our system is 0.5–1.0 m.

**Figure 2:**
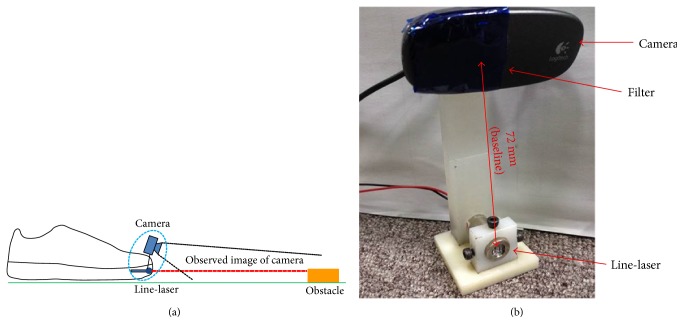
(a) System configuration of line-laser obstacle detection system integrated on shoes and (b) prototype of the proposed system.

In our prototype, a Logitech C310 webcam that operates at 29 frames per second under a 640 × 480 resolution and a 405 nm wavelength laser are used. Both of them are mounted rigidly to have consistent calibration parameters. Moreover, to suppress the noise interference, a blue glass paper is used as a band-pass filter to resist the unnecessary light from the environment, as shown in Figure 2.

### 3.2. Software Framework

**Figure 3:**
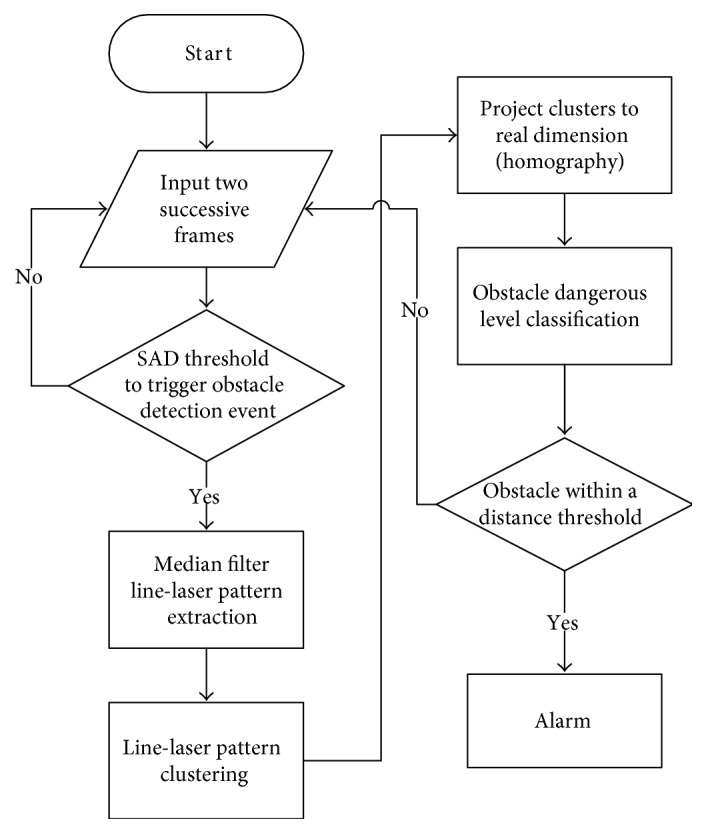
Software framework of line-laser obstacle detection system.

Our algorithm flowchart is shown in Figure 3. Initially, the camera continuously acquires an image and then the obtained image is compared with the previous one. If the difference between two successive frames is very small, we assume that the users step on the ground. Besides, the moment the users raise the foot to the max height during a gait cycle also has the same effect. In both the above cases, the event for determining the obstacles will be triggered. Otherwise, the program will only continuously calculate the difference between two incoming frames.

Once the obstacle detection event is triggered, a series of line-laser pattern extraction steps will be executed. First, the median filter is applied in order to suppress noises. In addition, an intensity threshold is used to identify the pixels where the laser light is. Then, the max value of each column of the image is extracted and considered as a potential laser pattern. After that, a segmentation method is applied to classify every laser pixel into several clusters that may denote the obstacles. Some clusters that do not represent obstacles are removed, and the clusters that denote the same obstacles together are merged. Subsequently, the real depths and widths of the obstacles are obtained by homography transformation. Finally, the system will send alarm messages according to the dangerous levels of the obstacles classified by their widths and depths. Each step in the software framework is illustrated in detail as follows.

### 3.3. Sum of Absolute Difference (SAD) Threshold to Trigger the Obstacle Detection Event

In order to detect the obstacles, the obstacle detection event is triggered when a user steps on the ground. Besides, to detect higher obstacles, the event is triggered when users raise their feet at the max height during a gait cycle as well. In both cases, their feet are roughly horizontal to the ground. Therefore, the system is suitable to detect the obstacles in front of the users at these moments. Besides, this strategy will save computation consumption at other times during a gait cycle.

The difference between the two frames that are captured by the camera is utilized to identify when the users either step on the ground or raise their feet at the max height. This concept was first proposed by Fitzpatrick and Kemp [19]. In our method, the normalized sum of absolute difference (SAD) value is utilized to compute the difference between two successive frames as in

(1) $$\begin{array}{c} SAD=\frac{\sum_{u=0}^{N_{rows}}\sum_{v=0}^{N_{cols}}\;|\;I_{p}\left(u,v\right)-I_{l}\left(u,v\right)\;|\;}{N_{rows}N_{cols}}, \end{array}$$

where *u* and *v* denote the pixel's coordinates on row and column directions. *N*_cols_ and *N*_rows_ are the pixel numbers of the image width and height. *I*_*p*_ and *I*_*l*_ represent the pixel intensity of the current frame and the previous frame, respectively. In general, the brightness of a color pixel comes from a linear combination of R, G, and B components. The intensity we used is only the B component. However, the intensity value of the camera highly depends on the environment illumination. Therefore, we turn off the auto white balance function and lock the exposure time for the camera.

**Figure 4:**
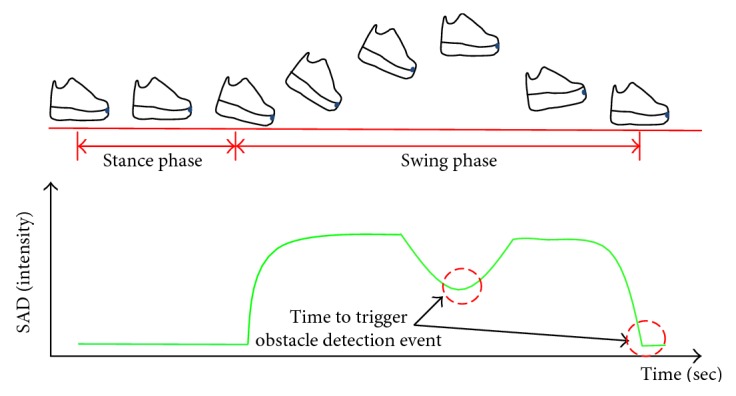
System trigger time determined by the SAD value.

In a gait, there are two phases: stance phase and swing phase. Usually, the SAD value between frames during a stance phase should be very small. Similarly, the SAD value will be small when the feet are at the max height. Once the users start to move their feet forward, the SAD value will become very large in contrast to the stance phase. In our experiment, the relatively small SAD value happens at the middle and at the end of every swing phase as shown in Figure 4. Therefore, the obstacle detection will be triggered when the SAD value is small.

### 3.4. Line-Laser Pattern Extraction

Once the obstacle detection event is triggered, a median filter is then applied to suppress the bad influences coming from the environmental noise [20]. The median filter with a specific window size sweeps across the whole image, and the intensity value of the middle pixel in the window will be replaced by the median of pixel values in the window. Therefore, it can maintain the original structure well, particularly for edge features.

After applying the median filter, an intensity threshold is set to separate the line-laser pattern from the background. At this step, any pixel with an intensity below the threshold is recognized as background or noise. On the other hand, if the intensity of a pixel exceeds the intensity threshold, it will be considered as a candidate pixel on the obstacles.

**Figure 5:**
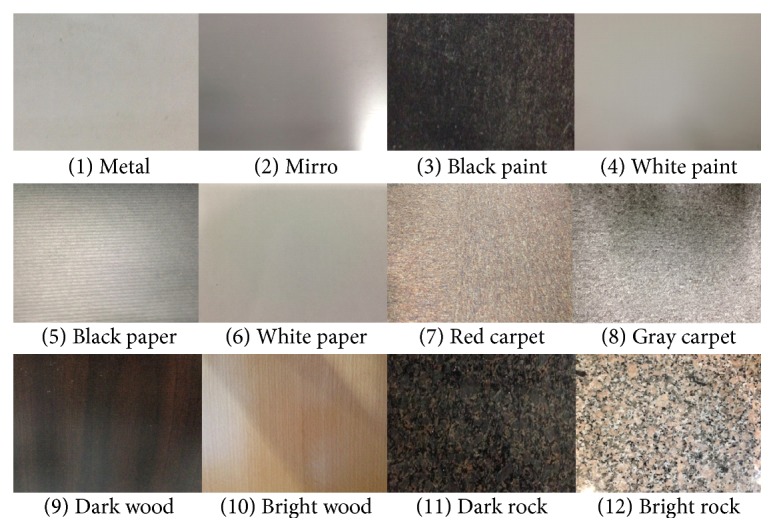
Common construction materials used indoors.

**Figure 6:**
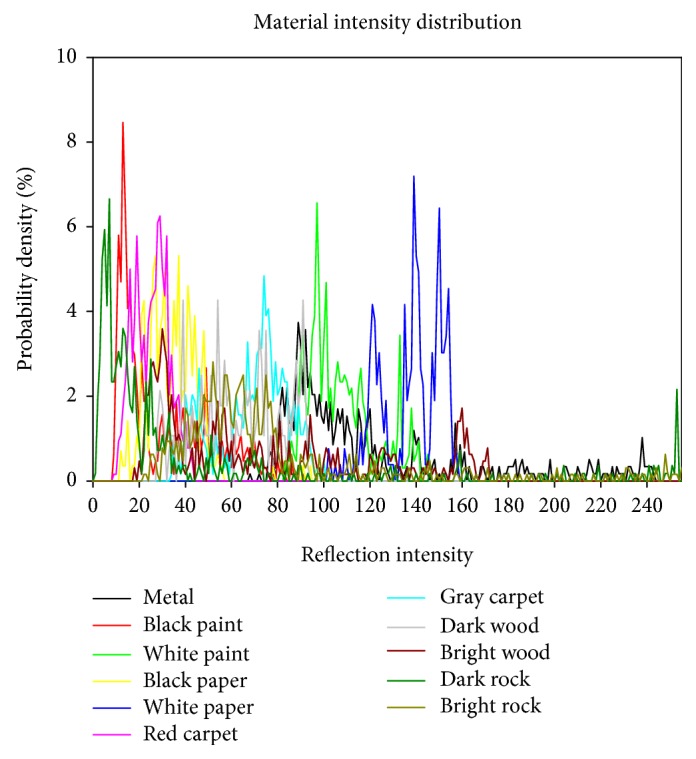
Reflection intensity distribution of common construction materials.

Since our system is mainly designed for indoor application, the intensity distribution of different obstacles that are detected by the system is needed to be investigated. Therefore, twelve common construction materials are surveyed, as arranged in Figure 5. After implementing detection experiment of each material, the intensity distribution data are depicted as shown in Figure 6, except that a mirror will directly reflect the laser light; therefore, it cannot be detected successfully by our system. In our application, the intensity threshold 14 is selected because 95% of the laser light among the experimental cases will pass the intensity threshold. On the contrary, if the intensity of any pixel is below the intensity threshold 14, it is recognized as a part of the background.

**Figure 7:**
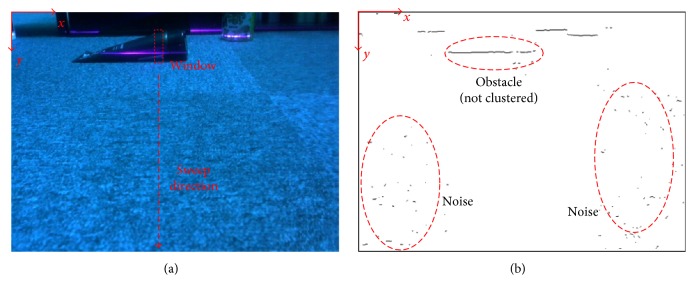
(a) Max average value of pixels in a window is searched to be the extracted line-laser pixel. (b) Line-laser pattern extraction result. The clusters in the red circle, which denote the same obstacle, break into parts, and some small clusters from the noise exist in the image.

Subsequently, a line-laser pattern is extracted by the following steps. The pixel having the max intensity value in each column is collected to be the line-laser pattern pixel. However, to increase the performance, the image is initially rotated 90° for processing and then rotated back. In addition, because of the need to reduce the impact of noise, the average of the pixel intensities within a window is searched for each column instead, as indicated in Figure 7(a). If the average value exceeds a specific threshold, the centroid of the window will be considered as the location of line-laser pattern in this column.

After the extraction of line-laser pattern, the extracted data are stored and processed again for obstacle clustering. Because of unstable line-laser intensities from the camera and disturbances from the environmental noise, the extracted line-laser pattern may suffer negative influence. In Figure 7(b), the example shows several noise groups and nonclustered obstacles. In these cases, noises will be rejected while the neighboring pixels are merged. In addition, a candidate obstacle will propagate due to the merging of neighboring clusters.

### 3.5. Line-Laser Pattern Clustering

The goal of our system is to recognize how far and how large the potential obstacles are. Therefore, a segmentation algorithm is needed after executing the line-laser pattern extraction procedure. This algorithm classifies each pixel on the line-laser pattern into several clusters that are likely denoting the obstacles.

**Figure 8:**
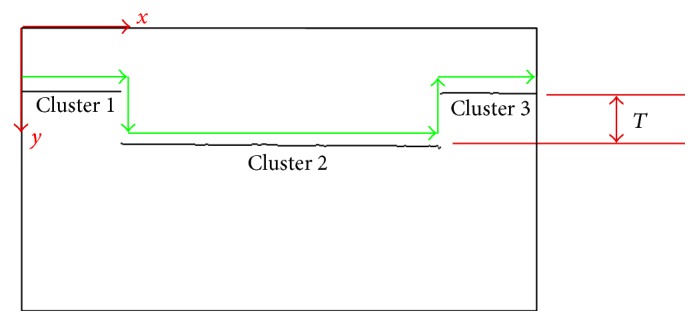
Search for line-laser pattern pixel by pixel for segmentation. If the *y* coordinate of the present pixel deviates from the last pixel and exceeds deviation threshold *T*, the present pixel is classified into another cluster.

We assume all pixels of an obstacle will form only a continuous segment. In condition, the distance deviation of the current cluster in the image domain will not exceed a specific threshold *T* as illustrated in Figure 8. Thus, a region-growing procedure is applied to one of unclassified pixels of the line-laser pattern. Therefore, several clusters that denote the obstacles are obtained.

A suitable threshold *T* in an indoor environment is critical. The experiment to determine *T* is described in a later section. Based on the discussion of the deviation threshold *T*, our deviation threshold for line segmentation is two pixels. Nevertheless, some clusters that belong to the same obstacle break apart, and they should be merged. We first eliminate small clusters that have very few pixels, less than six, and then merge the residuals by a deviation threshold.

**Figure 9:**
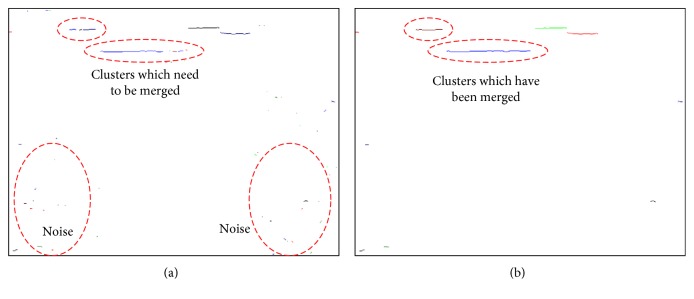
(a) Result of line-laser pattern clustering. Some residual noise needs to be further eliminated. Besides, clusters that belong to the same obstacle should be merged. (b) The result after executing noise illumination and merging.

In order to eliminate the small clusters from the environmental noise, the width of the segments, which come from the noise of images, are counted. The statistical result indicates that the widths of 95% clusters of noises are below six pixels. Therefore, clusters with widths less than or equal to six pixels are recognized as noises. Furthermore, clusters with less than six pixels will have a 0.45 cm of width in the middle of our working region. If the horizontal distance between two neighboring clusters is less than the width of a foot, people may still kick on the gap between two obstacles and then trip over. Therefore, on the condition that the *y* coordinate deviation on extracted line-laser image between two clusters is within the deviation threshold *T*, and the real world distance of the gap between two clusters is less than the width of a foot (10 cm), these two clusters should be merged. An example is illustrated in Figure 9(a). The merging step is taken from the leftmost cluster to the right. The result after executing noise elimination and the merging steps is shown in Figure 9(b).

### 3.6. Homography Transformation

**Figure 10:**
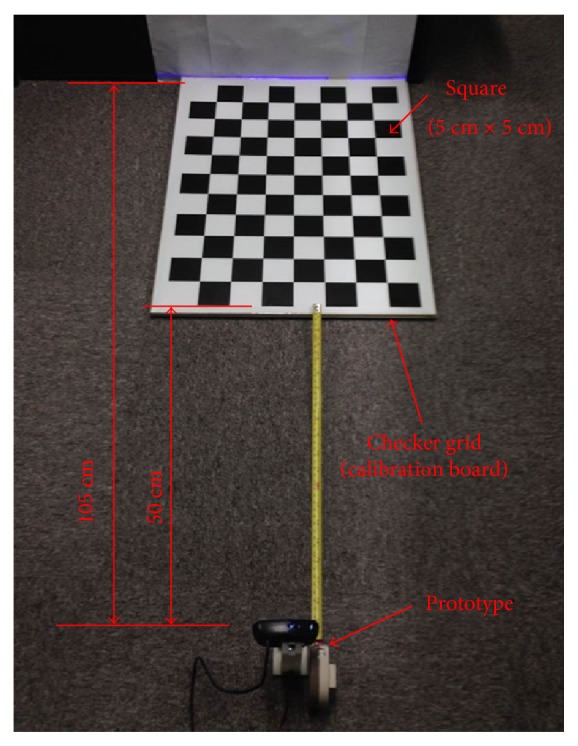
Calibration setup of our prototype.

After executing the line-laser pattern clustering, the physical distance is needed to be determined by homography transformation. The calibration for homography transformation in our paper utilizes a checkerboard. The transformation denotes the relationship between the image coordinate and the real world coordinate of the checker grid, as shown in Figure 10. Since the detection region of our prototype is 0.5–1.0 m, the obstacle detection does not need very accurate distance estimation. Therefore, the lens distortion of the camera in our prototype is negligible.

A coordinate [*x*, *y*, 1]^*T*^ on the image plane can be mapped into the real world coordinate [*x*_1_′, *x*_2_′, *x*_3_′]^*T*^, which indicates a homogeneous coordinate after applying a 3 × 3 homography matrix, as (2) [21]. The real world coordinate can be converted into a real dimension by (3).

(2) $$\begin{array}{c} \left[\begin{array}{c} x_{1}^{\prime }\\ x_{2}^{\prime }\\ x_{3}^{\prime } \end{array}\right]=\left[\begin{array}{ccc} h_{11} & h_{12} & h_{13}\\ h_{21} & h_{22} & h_{23}\\ h_{31} & h_{32} & h_{33} \end{array}\right]\left[\begin{array}{c} x\\ y\\ 1 \end{array}\right]. \end{array}$$

(3) $$\begin{array}{c} \left(x^{\prime },y^{\prime }\right)=\left(\frac{x_{1}^{\prime }}{x_{3}^{\prime }},\frac{x_{2}^{\prime }}{x_{3}^{\prime }}\right). \end{array}$$

**Figure 11:**
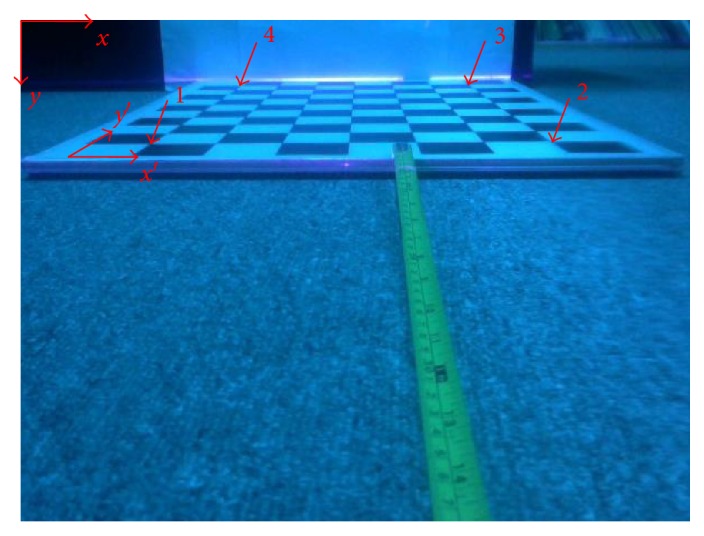
Four cross corners in real world coordinates and their corresponding points in the image coordinates are utilized to determine the homography transformation between two coordinates.

By rewriting (2) and (3), eight unknowns in the homography matrix become (4), where *h*_33_ = 1. Because there are eight unknowns to be solved, we need at least four corresponding points to solve the homogeneous matrix. In the calibration procedure, the calibration board is put in front of our prototype. In addition, the line-laser roughly passes through the surface of the calibration board. After taking a picture for the checker grid, four points on the image and their corresponding cross corners on the checker grid are utilized to determine the homography matrix, as shown in Figure 11. For example, four cross corners in real coordinate with centimeter will be (5, 5), (35, 5), (35, 50), and (5, 50) in sequence. By solving (4), eight unknowns representing a homography matrix are obtained. In practice, the real world coordinate should be shifted again due to a translation in the laser's position.

(4) $$\begin{array}{c} \left[\begin{array}{cccccccc} x & \;y & 1 & 0 & 0 & 0 & -x^{\prime }x & -x^{\prime }y\\ 0 & 0 & 0 & x & y & 1 & -y^{\prime }x & -y^{\prime }y \end{array}\right]\left[\begin{array}{c} h_{11}\\ h_{12}\\ h_{13}\\ h_{21}\\ h_{22}\\ h_{23}\\ h_{31}\\ h_{32} \end{array}\right]=\left[\begin{array}{c} x^{\prime }\\ y^{\prime } \end{array}\right]. \end{array}$$

### 3.7. Obstacle Alarm Level Classification

**Figure 12:**
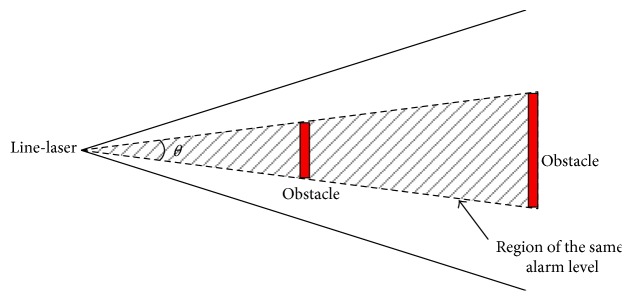
Obstacle alarm level of each obstacle can be classified by the angle *θ* in this figure. The two obstacles are both in the triangular shaded region, and thus, their alarm levels are the same.

As long as the width and the depth of each obstacle can be calculated, the obstacle alarm level of each obstacle can be classified. Since the size and distance may affect the dangerous level, we use a coverage angle *θ* to a normalized factor for alarm as shown in Figure 12. The two obstacles in Figure 12 are of the same coverage angle to the laser position, and thus, their alarm levels are considered to be the same. In our proposed system, alarm level classification is used because residual small clusters may cause the system to send false positive alarm messages on the condition that the system just determines whether dangerous obstacles exist.

The system distinguishes the obstacles into four alarm levels. Alarm level 1 means the obstacle is the most dangerous, causing the system to send the most urgent and loud alarm messages. On the other hand, when alarm level of the obstacles becomes 2 or 3, the alarm message will become weaker. Last, if the alarm level of the obstacles is 4, the system will not react and send any alarm messages.

## 4. Result and Performance Evaluation

### 4.1. SAD Threshold Determination

**Figure 13:**
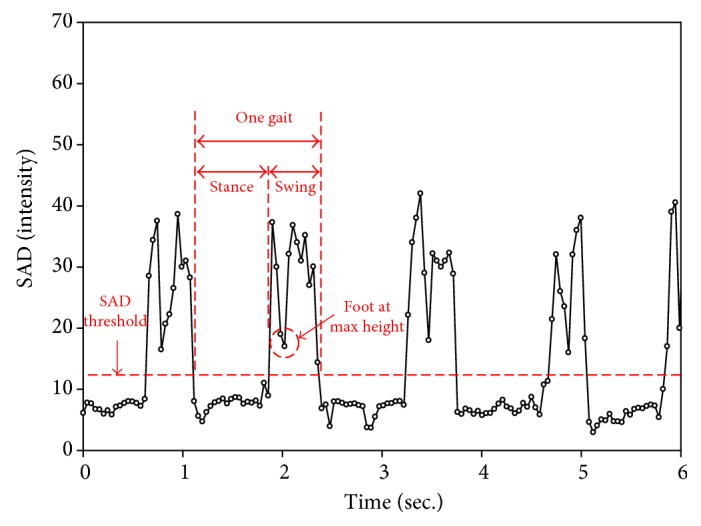
SAD values of several gaits. The threshold is determined below the average of SAD values in one gait.

In order to determine a proper SAD threshold for triggering the obstacle detection event, the camera continues recording SAD values during a gait cycle. A person wore the shoes and then walked around for a period of time. The collected data are shown in Figure 13. It is clear that a periodical shape comes out. After collecting the data, the SAD threshold, which represents the transition SAD value of the stance phase and swing phase, is obtained by averaging the SAD values of all the frames in one gait. A threshold equaling to 15.8 is therefore obtained. In Figure 13, the SAD value in the stance is stable and is as low as 8.2. To define a strict threshold, a threshold value of 12 is finally determined.

Apart from the moment a user steps on the ground, the obstacle detection event should be triggered when people raise their foot at max height to detect higher obstacles. However, a SAD threshold to determine the moment in swing phase is difficult to be identified because of the unsteady characteristics of different steps. Besides, it may vary in the gait behavior. Therefore, a reasonable definition for the moment when the elders' foot at max height highly depends on the collection of data. In our experiment, the moment is roughly at one third of a gait cycle, which has a relative small SAD value within the 3rd–7th frames during the swing phase. In practice, these five frames are temporarily stored, and the smallest SAD value is then selected as the moment for triggering obstacle detection event.

### 4.2. Deviation Threshold *T*

In obstacle detection event, we use a deviation threshold *T* for rejecting neighboring pixels that do not belong to the current segment. The deviation threshold value *T* for the line segmentation is critical. If the deviation threshold *T* is set at a value too small, one cluster may be classified into many different smaller clusters, and this may cause the original cluster to be neglected by our system. On the other hand, if the deviation threshold is chosen to be too large, some clusters that belong to different obstacles may be grouped together. As a result, the system will over emit alert signals to the users.

In our system, a line-laser casts light on the obstacles and one camera observes the line patterns. Therefore, the cast line becomes broader and stronger at a smaller distance due to the perspective effect. However, after analyzing the line-laser pattern at each distance, the deviation threshold *T* = 1 is the same for all distances in the detection region 0.5–1.0 m. In other words, a suitable deviation threshold *T* will not change with detection distance when the line-laser obstacle detection system detects the same obstacle.

On the other hand, the deviation of pixels of the same cluster varies and depends on the reflectivity of obstacles. Therefore, an experiment is designed to find out a suitable deviation threshold *T* of common construction materials that are usually used indoors. Twelve test samples are considered as shown in Figure 5. Because a suitable deviation threshold *T* will not vary with the detection distance, all the experiments were carried out at a distance of 100 cm. After the experiment, a conclusion is drawn that except the dark rock (sample number 11) that needs the deviation threshold *T* to be 2 pixels for correct segmentation and that the mirror (sample number 2) cannot be detected directly because of its high reflectivity, the suitable deviation threshold for the rest of the samples is 1 pixel. However, in order to handle all the cases strictly, the proper deviation threshold *T* is set to be 2 pixels for the correct operation of our line-laser obstacle detection system.

### 4.3. Distance Estimation by Homography

Furthermore, an experiment is carried out to validate the precision of the distance estimation by homography. The line-laser obstacle detection system is mounted on a linear slider rigidly, and then the linear slider is used to adjust the distance between our system and a flat wall. The distance computation result by homography mapping will be compared to the standard distance measurement by the slider. From the validation experiment, the measurement error at 80 cm, the middle of the working distance, is less than 0.3 cm. At the nearest working distance, say 50 cm, the measured error is 1.7 cm, as shown in Table 1. This error value is acceptable compared to the step lengths of the elderly.

### 4.4. Obstacle Alarm Level Classification Results

The line-laser obstacle detection system classifies the obstacle alarm levels according to their angles. We define four alarm levels. When the obstacle coverage angle *θ* > 7.8°, the alarm level is 1. That represents that an obstacle with a 10 cm width, as well as the foot width of an adult, is located as far at 75 cm in front of the user's shoes. The alarm level 2 is assigned when the angle *θ* is between 3.8° and 7.8°. Similarly, alarm level 3 is raised when the angle *θ* is between 1.5° and 3.8°. If the angle *θ* is less than 1.5°, which indicates that the obstacle's width is as small as 2 cm in the middle of the working distance, alarm level 4 is obtained and no alarm signal will be sent.

**Figure 14:**
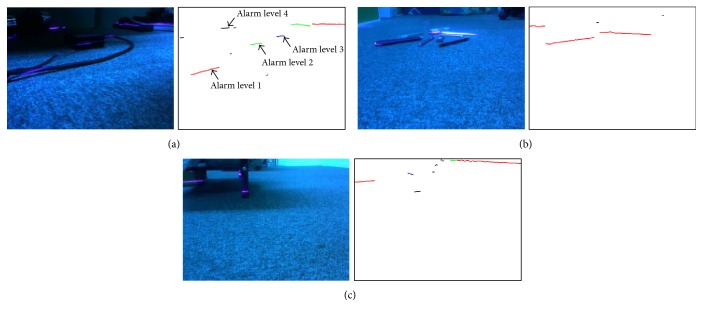
(a) System detects a cable on the ground. The red clusters represent alarm level 1, the green clusters represent alarm level 2, and the blue clusters represent alarm level 3. The black clusters denote alarm level 4. (b) System detects scatterings on the ground. (c) The system detects a corner of a table.

The system performance is tested by detecting real obstacles, which may occur regularly indoors, as shown in Figure 14. The experiment results prove that the system can work well to compute the widths and the distances of obstacles and then determine their alarm messages. In Figure 14(a), four cases are included and the highest priority, say level 1, will be finally sent. Based on this strategy, the probability of false positive errors will be low, and the alarm messages can be sent out according to the dangerous levels of encountered obstacles.

### 4.5. Limitations of the Proposed Method

With regard to the limitations of our method, the camera may suffer from the drawback of its low dynamic range. Besides the height of detectable, the obstacles must be larger than the plane level of the line-laser. Nevertheless, the feature of fall prevention is carried out by strategically sending an alarm message to the users.

## 5. Conclusion

In this paper, a camera-based line-laser obstacle detection system is proposed for designing fall prevention shoes of users. The system simply consists of an RGB camera, a filter, and a line-laser, so it is suitable to be installed on customer wearable devices, and the overall costs of the products are acceptable compared to shoes. We successfully verified the algorithms, including SAD threshold to trigger the obstacle detection event, line-laser pattern segmentation, homography transformation, and obstacle dangerous level classification. Finally, a prototype for the prevention of falls of the elderly was carried out.

## Figures and Tables

**Table 1:** Distance estimation.

Ground truth distance (cm)	Estimated distance (cm)	Error (cm)	Error (%)
50	51.7	1.7	3.4
60	61.1	1.1	1.8
70	70.4	0.4	0.5
80	79.8	−0.2	−0.3
90	88.9	1.1	−1.2
100	99.5	−0.5	−0.5
